# Draft Genome Sequences of *Geobacillus stearothermophilus* Strains 22 and 53, Isolated from the Garga Hot Spring in the Barguzin River Valley of the Russian Federation

**DOI:** 10.1128/genomeA.01205-14

**Published:** 2014-11-20

**Authors:** Aleksey S. Rozanov, Maria D. Logacheva, Sergey E. Peltek

**Affiliations:** a Institute of Cytology and Genetics, Siberian Branch of the Russian Academy of Sciences, Novosibirsk, Russian Federation; b Lomonosov Moscow State University, Moscow, Russian Federation

## Abstract

*Geobacillus stearothermophilus* strains 22 and 53 were isolated from sediment samples isolated from the Garga hot spring (72°C) located in the valley of the river Barguzin (the Baikal region, Russian Federation) (54°19′3.72″N, 110°59′38.4″E).

## GENOME ANNOUNCEMENT

In 2001, T. Nazina et al. described the genus *Geobacillus* and transferred six existing Bacillus species into it (*Geobacillus stearothermophilus*, *Geobacillus thermocatenulatus*, *Geobacillus thermoleovorans*, *Geobacillus kaustophilus*, *Geobacillus thermoglucosidasius*, and *Geobacillus thermodenitrificans*). They also proposed two new species for the genus: *Geobacillus subterraneus* and *Geobacillus uzunensis* (1). Subsequently, other new species were added to the genus *Geobacillus*: *Geobacillus toebii* (2), *Geobacillus debilis* (3), *Geobacillus lithuanicus* (4), and *Geobacillus gargensis* (5). The phylogeny of several other species is still uncertain (6).

*Geobacillus stearothermophilus* strains 22 and 53 were isolated from sediment samples isolated from the Garga hot spring (72°C) located in the valley of the river Barguzin (the Baikal region, Russian Federation) (54°19′3.72″N, 110°59′38.4″E).

For DNA isolation, bacterial culture was cultivated in a liquid medium containing 1% trypton and 0.5% yeast extract, and 8 mL of cell culture were pelleted by centrifugation and resuspended in 75 µL of H_2_O by intense pipetting. DNA preparations for genome sequencing were made using the GeneJET DNA purification kit (Fermentas) according to the manufacturer’s instructions. The Nextera DNA sample prep kit (Illumina) was used to create libraries for genome sequencing. Genomic DNA was sequenced using the MiSeq Reagent kit version 2 (Illumina) in the Laboratory of Evolutionary Genomics of the Faculty of Bioengineering and Bioinformatics, Moscow State University.

*De novo* assembly of short reads into contigs was performed using SPAdes v3.1.0. Contigs shorter than 1,000 bp were deleted. Open reading frame (ORF) prediction and automatic annotation was performed using NCBI PGAAP (http://www.ncbi.nlm.nih.gov/genome/annotation_prok). For strain 22, a total of 199 contigs yielded a genome sequence 3.27 Mb long, and the G+C content was 52.6%. The draft genome sequence contained 3,121 genes, 2,992 coding sequences (CDS), 19 rRNAs (5S, 16S, 23S), 62 tRNAs, and one noncoding RNA (ncRNA). For strain 53, a total of 178 contigs yielded a genome sequence 3.27 Mb long, and the G+C content was 52.6%. The draft genome sequence contained 3,196 genes, 3,042 CDS, 25 rRNAs (5S, 16S, 23S), 79 tRNAs, and one ncRNA.

Phylogenetic analysis was performed using 16S rRNA sequences with the UPGMA algorithm implemented in MEGA v6. 16S rRNA sequences of *Geobacillus* type strains were found using the StrainInfo (http://www.straininfo.net) and GenBank (http://www.ncbi.nlm.nih.gov/nucleotide) databases. According to phylogenetic analysis, strains 22 and 53 can be assigned to species *Geobacillus stearothermophillus*.

### Nucleotide sequence accession numbers.

The draft genome sequence for *Geobacillus stearothermophilus* strain 22 has been deposited at DDBJ/EMBL/Genbank under the accession number JQCS00000000. The 199 contigs have been deposited under the accession numbers JQCS01000001 to JQCS01000199. The draft genome sequence for *Geobacillus stearothermophilus* strain 53 has been deposited in DDBJ/EMBL/Genbank under the accession number JPYV00000000. The 178 contigs have been deposited under the accession numbers JPYV01000001 to JPYV01000178.
